# Personality, Socio-Economic Status and Inflammation: Cross-Sectional, Population-Based Study

**DOI:** 10.1371/journal.pone.0058256

**Published:** 2013-03-13

**Authors:** Keith Millar, Suzanne M. Lloyd, Jennifer S. McLean, G. David Batty, Harry Burns, Jonathan Cavanagh, Kevin A. Deans, Ian Ford, Alex McConnachie, Agnes McGinty, Réne Mõttus, Chris J. Packard, Naveed Sattar, Paul G. Shiels, Yoga N. Velupillai, Carol Tannahill

**Affiliations:** 1 College of Medical, Veterinary and Life Sciences, Institute of Mental Health and Wellbeing, University of Glasgow, Glasgow, Scotland; 2 Robertson Centre for Biostatistics, University of Glasgow, Glasgow, Scotland; 3 Glasgow Centre for Population Health, Glasgow, Scotland; 4 MRC Social and Public Health Sciences Unit, Glasgow, Scotland; 5 Department of Epidemiology and Public Health, University College London, London, England; 6 Scottish Government, Edinburgh, Scotland; 7 Department of Vascular Biochemistry, NHS Greater Glasgow and Clyde, Glasgow Royal Infirmary, Glasgow, Scotland; 8 Department of Clinical Biochemistry, NHS Grampian, Aberdeen Royal Infirmary, Aberdeen, Scotland; 9 Glasgow Clinical Research Facility, Tennent Building, Western Infirmary, Glasgow, Scotland; 10 Centre for Cognitive Ageing and Cognitive Epidemiology, Department of Psychology, University of Edinburgh, Edinburgh, Scotland; 11 Institute of Cardiovascular and Medical Sciences, BHF Glasgow Cardiovascular Research Centre, University of Glasgow, Glasgow, Scotland; 12 Institute of Cancer Sciences, College of Medical, Veterinary and Life Sciences, University of Glasgow, Glasgow, Scotland; 13 Graduate Entry Medical School, University of Limerick, Limerick, Ireland; University of Leicester, United Kingdom

## Abstract

**Background:**

Associations between socio-economic status (SES), personality and inflammation were examined to determine whether low SES subjects scoring high on neuroticism or hostility might suffer relatively higher levels of inflammation than affluent subjects.

**Methods:**

In a cross-sectional design, 666 subjects were recruited from areas of high (most deprived – “MD”) and low (least deprived – “LD”) deprivation. IL-6, ICAM-1, CRP and fibrinogen were measured along with demographic and health-behaviour variables, and personality traits of neuroticism, extraversion and psychoticism (hostility). Regression models assessed the prediction of inflammation as a function of personality, deprivation and their interaction.

**Results:**

Levels of CRP and IL-6 were an increasing function of neuroticism and extraversion only in LD subjects opposite trends were seen in MD subjects. The result was ascribed parsimoniously to an inflammatory ceiling effect or, more speculatively, to SES-related health-behaviour differences. Psychoticism was strongly associated with ICAM-1 in both MD and LD subjects.

**Conclusions:**

The association between neuroticism, CRP and IL-6 may be reduced in MD subjects confirming speculation that the association differs across population sub-groups. The association between psychoticism and ICAM-1 supports evidence that hostility has adverse effects upon the endothelium, with consequences for cardiovascular health. Health interventions may be more effective by accounting for personality-related effects upon biological processes.

## Introduction

Individuals who display certain personality characteristics are more likely to indulge in harmful health behaviours [1]–[8] and to have increased risk of morbidity and mortality [7], [9]–[11]. We have previously extended such findings to examine the association between personality, mental well-being and health behaviours as a function of socio-economic status (SES) [12]. Here, we consider the further association between SES, personality and inflammation.

There is growing evidence to link personality characteristics to inflammatory processes. For example, high levels of neuroticism (N) and low conscientiousness (C) have been associated with elevated levels of C-reactive protein (CRP) and Interleukin-6 (IL-6) [13], and “pessimistic worry” (a feature of neuroticism) has been linked to high levels of CRP [14]. Similar associations have been shown between high levels of hostility and CRP and IL-6 [15]. Higher levels of inflammation are often consequences of harmful health behaviours such as smoking, poor diet and lack of exercise, and it is significant that the latter behaviours are also characteristic of high N, low C and psychoticism (P) [1]–[6], [8]. However, the fact that the relationship between high N and mortality has been shown in one study to be independent of smoking and exercise [16] might imply the role of factors other than health behaviours. Most recently, one of our groups [17] has confirmed the negative relationship between C and CRP, and that the association was mediated by body mass index (BMI) but not by common health behaviours of smoking, physical activity and alcohol consumption. Moreover, whilst N has been associated with adverse effects on health [11], [16], a protective role for the factor has also been reported [18], [19] when neuroticism-related health anxiety leads to positive health behaviours that may have beneficial consequences for health [20].

An association between personality and inflammation might have particular implications for socially-disadvantaged individuals. Low SES is associated with high levels of N [21], [22], low levels of C [22], higher hostility [23] and depression [24]. In separate research, low SES was also associated with high levels of inflammatory markers that are in part consequences of harmful health behaviours and a stressful social environment associated with deprivation [25]–[28]. Given the latter associations, there may then be concern that deprived individuals having neurotic or hostile traits may be at risk of disproportionately greater levels of inflammatory activity. To the present authors’ knowledge, no study has considered the interaction between personality factors, SES and inflammation.

The present study examines the association between SES, personality and inflammation in a cohort recruited from the most affluent and most deprived areas of a large British city. The cohort comprises the Psychological, Social and Biological Determinants of Ill Health (“pSoBid”) [29], [30] study which provides a broad range of biological, social and psychological variables which permit evaluation of the association between personality, inflammation and SES. The database also allows adjustment for covariates such as depression and BMI that are associated with inflammation but which have been omitted from some previous investigations of personality and inflammation [31], and for the factor of intelligence which may influence mortality and morbidity [32]–[34] in part via the health behaviours that are also associated with high inflammation.

## Methods

### Ethical Approval

The study was approved by the Glasgow Royal Infirmary Research Ethics Committee. Participants gave their written informed consent.

### Study Population and Protocol

The design of the Psychological, Social and Biological Determinants of Ill Health (pSoBid) study has been described in detail elsewhere [29]. Briefly, selection of subjects was based on the Scottish Index of Multiple Deprivation (SIMD) 2004 [35] which rank small areas on the basis of multiple deprivation indicators. Subjects were recruited from five general medical practices in the city of Glasgow, Scotland, that served the bottom 5% of SIMD (i.e. relatively deprived) and five practices in areas classified as the top 20% of the SIMD (i.e. relatively affluent). Between December 2005 and May 2007 we recruited approximately equal numbers from both areas, equal numbers of men and women and equal numbers from each age group (35–44, 45–54 and 55–64 years old).

The original focus of the study was upon factors explaining the marked SES gradient in health, and in particular the role of inflammatory processes and their associated consequences for cardiovascular function. Sample size was therefore determined by the numbers required to detect differences between deprived and affluent groups in mean CRP levels (84% power to detect a 30% difference) and carotid intima media thickness (c-IMT: 82% power to detect a 0.04 mm difference), and did not take specific account of the small effect sizes associated with personality variables [13]: this issue is considered further below.

### Inflammatory Markers

High sensitivity C-reactive protein (CRP) was measured by an immunoturbidimetric assay (Roche Diagnostics Ltd., Burgess Hill, United Kingdom). Interleukin-6 (IL-6) and Intercellular Adhesion Molecule-1 (ICAM) were measured by sandwich ELISA (R&D Systems Europe Ltd., Abingdon, United Kingdom). Fibrinogen was measured on an automated coagulometer (MDA-180, Organon Teknika, Cambridge, United Kingdom).

### Psychological, Lifestyle and Clinical Assessment

The short scale of the Eysenck Personality Questionnaire [36] assessed neuroticism (N), psychoticism (P) and extraversion (E) and included a “lie” scale to detect those seeking to present themselves in a socially ideal light. The National Adult Reading Test [37] (NART) defined the individual’s peak achieved level of intellectual function [38], or pre-morbid intelligence [39]: the error score correlates negatively with intellectual function or “IQ”. The depression sub-scale of the General Health Questionnaire [40] measured low mood. A lifestyle questionnaire assessed health-related variables including regular exercise, alcohol intake, dietary score [12] and smoking. In addition, subjects attended for a detailed clinical assessment which included blood pressure, body mass index (BMI) and c-IMT measurement [29].

### Statistical Analysis

Descriptive statistics for continuous variables are presented as mean and standard deviation (SD) or median and interquartile range (IQR), as appropriate, and for categorical variables as frequencies and percentages.

Baseline differences between the groups were tested using linear and (binary or ordinal) logistic regression models for continuous and categorical variables respectively and adjusted for age and sex.

Inflammatory markers were tested for associations with the variables of age, gender, deprivation group, current smoker, alcohol consumption, physical activity, diet score, BMI, GHQ depression score, years of education and NART error score in separate linear regression models. Logarithmic transformations were applied to CRP, IL-6 and ICAM-1. Results are reported as the estimated regression coefficient, its 95% confidence interval (CI) and p-value.

Associations between each inflammatory marker and N, E and P were also tested with linear regression models which included terms for main effects and their interactions with deprivation. Results are reported as the effect estimate for the personality factor (with 95% CI and p-value) within each group, and the p-value for the interaction term testing whether the separate associations are different.

Then, for each inflammatory marker, all the predictor variables were entered into a backward stepwise regression; for the personality factors, the starting model included terms for interactions with deprivation. Terms were removed if they did not improve the fit of the model at a 5% significance level (subject to not removing the main effects of deprivation or a psychological variable whilst their interaction remained in the model). Those factors remaining in the final model are reported as above. This process was repeated, starting with factors found to be significantly associated with the inflammatory marker on univariate analysis, which gave the same results in each case. For those personality measures that were associated with inflammatory markers, the results are also presented graphically as the predicted level of each marker in relation to the personality variable, with a 95% CI, in the most and least deprived groups.

In addition, we examined whether the LD and MD groups differed in their associations between E and N and health behaviours (smoking, alcohol consumption, diet and physical exercise), and other salient factors of BMI, GHQ depression scores, years of education and NART error scores.

## Results

Table 1 confirms that the least deprived (LD) and most deprived (MD) groups were well matched on age and gender, but differed on a range of socioeconomic and lifestyle characteristics. Table 1 also shows, as expected, higher levels of CRP, IL-6, ICAM-1 and fibrinogen in the MD group, as well as higher scores on N and P compared to the LD group (adjusted for age and sex). The groups did not differ significantly in mean E or “Lie” scores.

**Table 1:** Description of basic demographics, socioeconomic status, personality and markers of inflammation by area deprivation category.

	Least Deprived (n = 342)	Most Deprived (n = 324)	p^b^	
Age (years)	51.8 (8.0)^a^	51.5 (8.5)	0.63	
Gender				
Male	171 (50.0%)	156 (48.1%)	0.64	
Female	171 (50.0%)	168 (51.9%)		
Household income	£41,699 (11,921)	£16,461 (10,056)	<0.001	
Education (total years)	16.1 (3.6)	11.8 (2.5)	<0.001	
Residential status				
Owner	334 (97.7%)	97 (29.9%)	<0.001	
Tenant	8 (2.3%)	227 (70.1%)		
Occupation category^c^				
I & II	251 (73.4%)	62 (19.1%)	<0.001	
III	77 (22.5%)	139 (42.9%)		
IV & V	12 (3.5%)	105 (32.4%)		
Unemployed	1 (0.3%)	2 (0.6%)		
Current Smoker				
Yes	28 (8.2%)	161 (49.7%)	<0.001	
No	314 (91.8%)	163 (50.3%)		
Alcohol (weekly units)	11.1 (12.7)	11.1 (21.5)	0.906	
Diet score^d^	95.7 (51.4)	59.9 (50.4)	<0.001	
Activity^e^				
Moderately Active – Active	176 (51.5%)	127 (39.2%)	<0.001	
Moderately Inactive – Inactive	166 (48.5%)	197 (60.8%)		
Body mass index (BMI)	26.9 (4.5)	28.7 (6.3)	<0.001	
Inflammatory Biomarkers				
C-reactive protein (CRP) (mg/l)^f^	1.09 [0.51–2.27]	2.12 [1.07–4.32]	<0.001	
Interleukin-6 (IL-6) (pg/ml)^f^	1.25 [0.87–1.95]	2.28 [1.34–3.13]	<0.001	
Intercellular adhesion molecule(ICAM-1) (ng/ml)^f^	229.4 [207.5–263.6]	297.8 [241.9–391.7]	<0.001	
Fibrinogen (g/l)	3.23 (0.64)	3.50 (0.76)	<0.001	
Eysenck Personality scores (EPQ-R)^g^				
Neuroticism (N)	4.06 (3.19)	5.96 (3.79)	<0.001	
Extraversion (E)	7.49 (3.41)	7.34 (3.61)	0.558	
Psychoticism (P)	1.26 (1.30)	2.58 (2.02)	<0.001	
Lie scale	5.35 (2.68)	5.34 (2.78)	0.962	
GHQ depression score^h^	0.17 (0.79)	0.76 (1.71)	<0.001	
NART error score^i^	7.16 (5.27)	15.61 (9.01)	<0.001	

a Values are presented as Mean (SD) for all participants; or as percentages for categorical variables, adjusted for age and sex;

b *P* relates the comparison between the two groups. Categorical variables were compared using Fisher’s exact test, continuous variables were compared using t-tests or Wilcoxon tests as appropriate.

c The occupational category could not be determined for n = 1 (0.3%) and n = 16 (5%) of the LD and MD groups respectively. Occupation classified using Registrar General Social Class Classification on basis of current job or, if not currently working, on the basis of participants’ last paid job. Only those who had never been in paid employment were classed as “unemployed.” I - professional occupations; II - managerial and technical occupations; III - manual and non-manual skilled occupations; IV - partly skilled occupations; V - unskilled occupations.

d Diet score is the participants self-reported consumption of fruit and vegetables (fresh, cooked and raw) per month.

e Physical activity level is a combination of activity at work and recreational exercise.

f Data log transformed prior to regression analysis.

g Personality trait scores were self-reported, each on a scale of 1 to 12.

h Depression = depression sub-scale of GHQ-28.

i NART (National Adult Reading Test).

### Personality, SES and Inflammation

Tables 2a to 2d show the outcome of analyses to examine the associations between the inflammatory markers and the above-listed independent variables, allowing for possible interactions with deprivation group.

**Table 2:** Univariate and multivariate associations between inflammatory markers and health-related variables, personality traits and deprivation grouping, and the interaction of personality and deprivation.

Independent variables	Univariate analysis			Multivariate analysis				
	Estimate (95% CI)	p	p_interaction_	Estimate (95% CI)	p		p_interaction_	
**2a Interleukin-6 (IL-6)**								
Deprivation status (MD vs LD)	0.428 (0.326, 0.529)	<0.001		0.205 (0.098, 0.311)	<0.001			
Age	0.221 (0.159, 0.284)	<0.001		0.192 (0.137, 0.247)	<0.001			
Gender (Male vs. Female)	0.053 (−0.053, 0.160)	0.327						
Education (total years)	−0.038 (−0.052, −0.024)	<0.001						
Current smoker (Yes vs. No)	0.392 (0.277, 0.508)	<0.001		0.385 (0.268, 0.502)	<0.001			
Alcohol (units per week)	0.002 (−0.001, 0.005)	0.252						
Diet score	−0.002 (−0.003, −0.001)	<0.001						
Activity (Active vs. Inactive)	−0.224 (−0.329, −0.118)	<0.001						
BMI	0.048 (0.039, 0.057)	<0.001		0.046 (0.037, 0.055)	<0.001			
GHQ Depression score	0.067 (0.026, 0.108)	0.001						
NART error score	0.018 (0.012, 0.024)	<0.001						
Neuroticism (N)	MD: −0.019 (−0.039, 0.001)	0.058	0.003	MD: −0.022 (−0.040, −0.005)	0.012		0.001	
	LD: 0.026 (0.004, 0.048)	0.020		LD: 0.021 (0.001, 0.040)	0.035			
Extraversion (E	MD: −0.008 (−0.029, 0.013)	0.436	0.215					
	LD: 0.010 (−0.011, 0.031)	0.329						
Psychoticism (P)	MD: 0.034 (−0.002, 0.071)	0.067	0.568					
	LD: 0.015 (−0.039, 0.069)	0.581						
**2b C-reactive protein (CRP)**								
Deprivation status (MD vs LD)	0.581 (0.411, 0.750)	<0.001		0.102 (−0.107, 0.311)		0.339		
Age	0.225 (0.120, 0.331)	<0.001		0.173 (0.077, 0.269)		<0.001		
Gender (Male vs. Female)	−0.138 (−0.313, 0.038)	0.123						
Education (total years)	−0.079 (−0.102, −0.056)	<0.001		−0.027 (−0.054, −0.001)		0.040		
Current smoker (Yes vs. No)	0.364 (0.170, 0.559)	<0.001		0.369 (0.166, 0.572)		<0.001		
Alcohol (units per week)	−0.003 (−0.009, 0.002)	0.176						
Diet score	−0.002 (−0.004, −0.0003)	0.021						
Activity (Active vs. Inactive)	−0.228 (−0.403, −0.053)	0.011						
BMI	0.089 (0.075, 0.104)	<0.001		0.087 (0.072, 0.102)		<0.001		
GHQ Depression score	0.129 (0.063, 0.195)	<0.001						
NART error score	0.033 (0.023, 0.043)	<0.001						
Neuroticism (N)	MD: 0.005 (−0.028, 0.038)	0.779	0.012	MD: −0.016 (−0.050, 0.018)		0.351		0.004
	LD: 0.069 (0.032, 0.106)	<0.001		LD: 0.054 (0.020, 0.089)		0.002		
Extraversion (E)	MD: −0.027 (−0.062, 0.008)	0.132	0.014	MD: −0.021 (−0.057, 0.014)		0.240		0.044
	LD: 0.035 (0.000, 0.070)	0.048		LD: 0.028 (−0.004, 0.060)		0.090		
Psychoticism (P)	MD: 0.043 (−0.019, 0.105)	0.173	0.809					
	LD: 0.029 (−0.062, 0.121)	0.529						
**2c. Intercellular Adhesion Molecule-1 (ICAM-1)**								
Deprivation status (MD vs LD)	0.248 (0.210, 0.287)	<0.001		0.079 (0.031, 0.128)		0.001		
Age	0.033 (0.007, 0.059)	0.013						
Gender (Male vs. Female)	0.006 (−0.037, 0.050)	0.768						
Education (total years)	−0.025 (−0.030, −0.020)	<0.001		−0.007 (−0.013, −0.002)		0.011		
Current smoker (Yes vs. No)	0.309 (0.267, 0.351)	<0.001		0.243 (0.196, 0.290)		<0.001		
Alcohol (units per week)	0.000 (−0.001, 0.001)	0.944						
Diet score	−0.001 (−0.002, −0.001)	<0.001						
Activity (Active vs. Inactive)	−0.082 (−0.125, −0.039)	<0.001						
BMI	0.006 (0.002, 0.010)	0.002		0.006 (0.003, 0.010)		<0.001		
GHQ Depression score	0.037 (0.021, 0.054)	<0.001						
NART error score	0.009 (0.007, 0.012)	<0.001						
Neuroticism (N)	MD: 0.003 (−0.005, 0.010)	0.443	0.982					
	LD: 0.003 (−0.006, 0.011)	0.508						
Extraversion (E)	MD: −0.006 (−0.014, 0.002)	0.148	0.100					
	LD: 0.004 (−0.004, 0.012)	0.376						
Psychoticism (P)	MD: 0.022 (0.009, 0.036)	0.002	0.947	All: 0.016 (0.006, 0.027)		0.002		
	LD: 0.023 (0.003, 0.044)	0.026						
**2d. Fibrinogen**								
Deprivation status (MD vs LD)	0.265 (0.155, 0.375)	<0.001						
Age	0.167 (0.100, 0.233)	<0.001		0.142 (0.079, 0.205)		<0.001		
Gender (Male vs. Female)	−0.136 (−0.247, −0.025)	0.017		−0.141 (−0.244, −0.038)		0.007		
Education (total years)	−0.029 (−0.043, −0.014)	<0.001						
Current smoker (Yes vs. No)	0.0.252 (0.128, 0.376)	<0.001		0.340 (0.222, 0.457)		<0.001		
Alcohol (units per week)	−0.002 (−0.005, 0.001)	0.256						
Diet score	0.000 (−0.001, 0.001)	0.890						
Activity (Active vs. Inactive)	−0.103 (−0.215, 0.008)	0.069						
BMI	0.039 (0.029, 0.049)	<0.001		0.041 (0.031, 0.050)		<0.001		
GHQ Depression score	0.054 (0.011, 0.097)	0.014						
NART error score	0.013 (0.006, 0.019)	<0.001						
Neuroticism (N)	MD: 0.001 (−0.020, 0.022)	0.943	0.089					
	LD: 0.028 (0.005, 0.052)	0.018						
Extraversion (E)	MD: −0.020 (−0.043, 0.002)	0.074	0.246					
	LD: −0.002 (−0.024, 0.021)	0.882						
Psychoticism (P)	MD: 0.027 (−0.013, 0.067)	0.182	0.523					
	LD: 0.004 (−0.054, 0.063)	0.890						

The outcome of initial univariate analysis shows associations between inflammatory marker and independent variables including the interactions of neuroticism (N), extraversion (E) and psychoticism (P) with deprivation status. Multivariate model determined by backward stepwise regression.

a BMI (body mass index).

b Depression = depression sub-scale of GHQ-28.

c NART (National Adult Reading Test).

**Figure 1:**
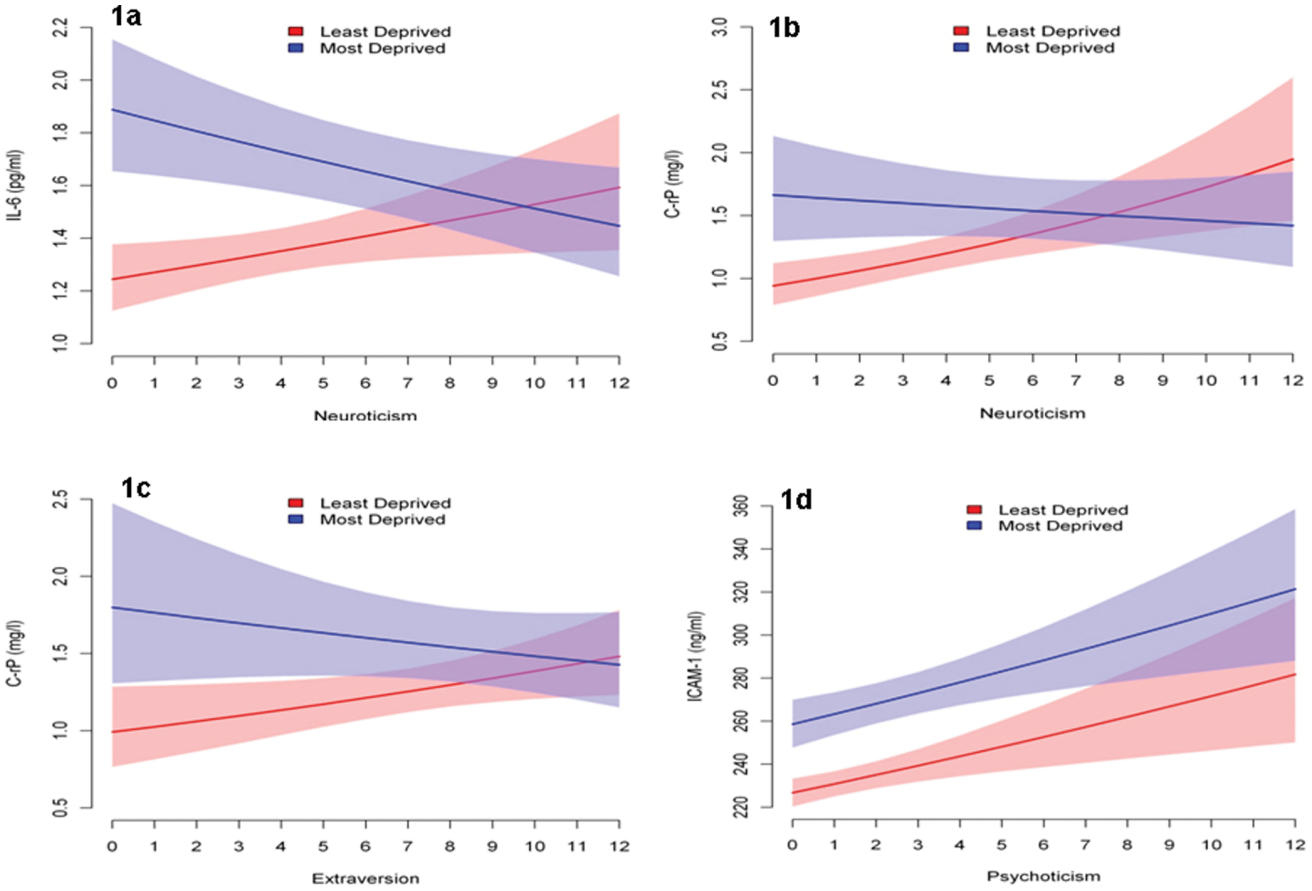
Levels of inflammatory markers as a function of the interaction between personality and deprivation group with 95% confidence bands.

IL-6 showed significant univariate associations with deprivation, greater age, current smoking, poorer diet, less physical exercise, greater BMI, higher depression GHQ score, fewer years of education and NART error score (denoting relatively lower intellectual function: Table 2a). With respect to personality, only N showed a univariate association with IL-6, the significant positive relationship implying that levels of the marker were an increasing function of N score across the sample. However, further analysis as a function of deprivation grouping showed that the positive relationship was true only in the LD group (p = 0.02) whilst a non-significant trend to a negative relationship was seen in MD subjects (p = 0.058) so that the interaction between N and deprivation grouping was significant (p = 0.003). These relationships, and the interaction, were confirmed in the multivariate analysis (Table 2a) and are illustrated in Figure 1a. The figure shows that whilst levels of IL-6 were overall higher in MD subjects they declined significantly as a function of N in contrast to the positive function seen in LD subjects so that there was therefore no disproportionate increase in levels of the marker amongst high-N-scoring MD subjects. The multivariate analysis also showed that deprivation grouping, current smoking and BMI remained significant predictors of IL-6.

CRP showed a similar interaction between N and deprivation grouping as in the case of IL-6 but without evidence of a significant negative association between the marker and N in the MD subjects (Table 2b). Multivariate analysis showed that whilst levels of CRP were overall higher in MD subjects, the between-group difference was not significant and that levels of the marker were an increasing function of N only in LD subjects (p = 0.002) so that the interaction between N and deprivation grouping was significant (p = 0.004). As shown in Figure 1b, there was again no evidence of a disproportionate increase in levels of the marker in high-N-scoring MD subjects. CRP was also associated with greater age, fewer years of education, current smoking and BMI.

The multivariate analysis also showed that neither the positive association between E and CRP in LD subjects, nor the opposite relationship in MD subjects was significant (p = 0.090 and p = 0.240, respectively: Table 2b and Figure 1c), although the interaction between E and deprivation grouping did reach significance (p = 0.044).

ICAM-1 (Table 2c) showed univariate associations with all variables except gender and alcohol consumption. In the multivariate analysis, only deprivation group, BMI, fewer years of education and current smoking remained significant. P was the only personality variable to be significantly associated with ICAM-1 in both the univariate and multivariate analyses, and with an equivalent positive association in both groups indicating that hostile and aggressive characteristics were associated with higher levels of the marker (Figure 1d).

In univariate analyses, fibrinogen (Table 2d) was associated with all variables except alcohol, diet and physical activity. Only age, gender, smoking and BMI were independently predictive in the multivariate model. In univariate analyses, whilst N was positively associated with fibrinogen in the population as a whole, no association was found in the multivariate model.

Whilst higher-order interactions have been shown between N and E in studies of the three-factor model [41] where, for example, high E scores might moderate the effects of high N, the present study found no such interactions.

Table 3 shows associations between the personality factors and health behaviours, and BMI, GHQ depression score, years of education and NART error score for MD and LD groups, and the interaction. E was positively associated with the NART error score in LD subjects but negatively associated in MD subjects so that the interaction was significant (p = 0.032). The factor of E was also strongly associated with fewer years of education only in the LD group so that the interaction was significant (p = 0.010). It is then evident that higher E scores in LD subjects were associated with lower intellectual status and fewer years of education relative to the associations seen in their MD counterparts. In the case of N, a similar but much weaker pattern of associations was observed so that only in the case of years of education was the interaction marginally significant (p = 0.058). Further weak associations were seen in the case of health behaviours. Table 3 shows that E was associated with a relatively greater propensity to take physical exercise in MD subjects (interaction: p = 0.090), with lower BMI (interaction: p = 0.055) and with a higher diet score. Strongest, and contrasting, associations were seen in the case of depression scores: in MD subjects, E was strongly associated with lower depression scores whilst N was strongly associated with higher scores (both interactions: p<0.001).

**Table 3:** Associations between personality traits and health behaviours in affluent and deprived groups, and their interactions.

	Least deprived	Most deprived	P Interaction
**Years of education**			
Neuroticism	−0.182 (−0.286, −0.078)	−0.050 (−0.142, 0.042)	0.058
Extraversion	−0.178 (−0.276, −0.081)	0.004 (−0.093, 0.100)	0.010
Psychoticism	−0.104 (−0.361, 0.154)	−0.072 (−0.246, 0.101)	0.840
**Current smoker (Yes vs. No)**			
Neuroticism	0.957 (0.842, 1.086)	1.048 (0.985, 1.116)	0.200
Extraversion	1.026 (0.911, 1.154)	0.981 (0.921, 1.045)	0.518
Psychoticism	1.317 (1.007, 1.724)	1.061 (0.945, 1.191)	0.144
**Alcohol (weekly units)**			
Neuroticism	−0.259 (−0.841, 0.323)	−0.221 (−0.736, 0.294)	0.923
Extraversion	0.565 (0.031, 1.100)	0.672 (0.149, 1.195)	0.779
Psychoticism	0.477 (−0.926, 1.879)	0.951 (0.006, 1.896)	0.577
**Diet score (fruit and vegetable consumption per month)**			
Neuroticism	−0.206 (−1.954, 1.542)	−1.460 (−3.005, 0.085)	0.283
Extraversion	1.078 (−0.526, 2.683)	2.431 (0.861, 4.000)	0.237
Psychoticism	−1.323 (−5.522, 2.877)	−0.987 (−3.817, 1.843)	0.895
**Activity (Active vs. Inactive)**			
Neuroticism	0.992 (0.925, 1.063)	0.950 (0.892, 1.012)	0.363
Extraversion	1.007 (0.944, 1.074)	1.091 (1.021, 1.167)	0.090
Psychoticism	0.993 (0.839, 1.177)	0.852 (0.749, 0.969)	0.151
**BMI** a			
Neuroticism	0.181 (−0.007, 0.368)	0.077 (−0.090, 0.243)	0.406
Extraversion	0.142 (−0.032, 0.317)	−0.097 (−0.268, 0.074)	0.055
Psychoticism	0.379 (−0.079, 0.838)	−0.068 (−0.378, 0.241)	0.109
**GHQ depression score** b			
Neuroticism	0.053 (0.012, 0.094)	0.184 (0.148, 0.220)	<0.001
Extraversion	−0.019 (−0.060, 0.022)	−0.128 (−0.168, −0.088)	<0.001
Psychoticism	0.037 (−0.068, 0.143)	0.054 (−0.018, 0.126)	0.792
**NART error score** c			
Neuroticism	−0.035 (−0.272, 0.202)	0.083 (−0.127, 0.294)	0.455
Extraversion	0.168 (−0.048, 0.383)	−0.162 (−0.373, 0.049)	0.032
Psychoticism	−0.241 (−0.805, 0.322)	0.434 (0.053, 0.814)	0.049

a BMI (body mass index).

b Depression = depression sub-scale of GHQ-28.

c NART (National Adult Reading Test).

### Summary of Principal Findings

Levels of inflammatory markers were overall higher in MD subjects, as were scores on N and P. However, levels of IL-6 and CRP were an increasing function of N only in LD subjects so that there was no disproportionate increase in the latter inflammatory markers in the MD group. A similar but weaker effect was seen between E and CRP. In the case of ICAM-1, the significant positive association between the marker and P was equivalent in both groups. In MD subjects, higher scores on E were associated with higher intellectual status, whilst an opposite relationship was seen in LD subjects.

## Discussion

The results confirm the established separate associations between low SES and high inflammation, and between low SES and high scores on N and P [15], [21]–[28]. Furthermore, to our knowledge, this study is the first to show that the relationship between personality and IL-6, and CRP, differs as a function of SES. Levels of the inflammatory markers were overall higher in the MD group, but as N scores increased, they were associated with increased levels of the marker only in the LD group. There was therefore no evidence that MD subjects having high N scores might suffer disproportionately higher levels of inflammation. A similar, although much weaker, association was observed between E and CRP.

A parsimonious explanation for these effects would be that, at high levels of inflammation, there is simply little scope for the known small and subtle influences of personality upon inflammation [13] to exert a detectable further increase in marker levels: in other words, a ceiling effect. However, whilst plausible, this proposal does not address the evidence that levels of IL-6 and, to a lesser extent, CRP declined as a function of high N in the MD group and with a similar trend being seen in the case of E and CRP.

The latter apparently beneficial effect of high N upon inflammation might be explained if neuroticism-related health anxiety exerted a protective effect through the adoption of positive health behaviours [17]–[19]. However, as no measure of health anxiety was included in the present methodology, this proposal cannot be assessed; nor is there obvious reason why these MD subjects might have been more prone to health anxieties. Moreover, there was no evidence in Table 3 of any significant associations between N and health behaviours in either of the two groups.

An alternative explanation may be found in differences between the groups in associations between certain health-related factors and N and E that might influence levels of inflammation. Fundamental individual characteristics that might exert such an influence would be those of intellectual status and years of education. Intellect is known to promote health by influencing self-care and health awareness [43], for example by taking greater dietary care and more exercise to the benefit of BMI, and smoking cessation. The latter factors are known to influence levels of inflammatory activity. Longer years of education may benefit health for similar reasons although the effects are less clear-cut [44]. The analysis showed that high E was associated with relatively higher intellectual function in MD subjects, whilst the opposite association was seen in the LD group. The high-E-scoring LD subjects also had fewer years of education and a similar although weaker association in LD subjects was seen in the case of N. A relatively greater intellect and longer years of education would plausibly tend to foster positive health behaviours. However, whilst high E in MD subjects was associated in Table 3 with a greater propensity to engage in physical activity, a lower BMI and a higher diet score than in high-E-scoring LD subjects, the associations were not formally significant. Moreover, as no adjustments were made in Table 3 for multiple statistical comparisons there is scope for Type-1 error: all p values must be considered as descriptive measures of strength of evidence for the observed associations. E scores were also associated with lower depression in MD subjects, but N scores showed an opposite relationship. The evidence that N was associated with higher depression scores in MD subjects would seem inconsistent with the motivation and drive to adopt positive health behaviours.

The analysis therefore provided some evidence that a number of health-related factors relevant to inflammation had different associations with E and N in MD and LD subjects. It must be speculative given the caveats above, but such differences may contribute in part to the contrasting associations seen between inflammation and personality in the two groups. Other relevant factors might be those of individual variation in coping strategies and resilience to socio-economic adversity, but we omitted to assess such characteristics.

More generally, the results parallel those of Chapman et al. [31] who reported no effect of N, and an inverse relationship between E and IL-6, in their study involving predominantly low SES subjects (their design precluded examining effects in higher SES subjects). The reduction in IL-6 was associated with the activity facet of extraversion (assessed by the NEO-FFI [42]) but we could not confirm this specific association due to limitations of the EPQ. Our findings have the advantage that the supposed low-SES-related association can be confirmed by comparison with an affluent sub-group. Overall, such results are consistent with proposals that the effects of personality may be absent, or of a different nature, in low SES groups and those of ethnic minorities [21], [31], leading to speculation that some traits of N and E may be associated with inflammation only in population sub-groups [45].

The significant association between P and ICAM-1 in both the deprived and affluent groups confirms existing evidence of an adverse influence of hostility upon endothelial function [46], and that cynical hostility is related to greater cytokine production [47] which, significantly in view of the present results, has been shown independent of adjustment for covariates including SES [48]. Our result would confirm that individuals high in hostility and aggression are at increased risk of inflammatory disease [15] and that this vulnerability is evident across the SES continuum.

Our failure to find a significant multivariate association between fibrinogen and the personality factors assessed here is similar to a recent negative result of Mõttus et al. [17] who note that fibrinogen is implicated in coagulation and is therefore a more peripheral and less sensitive inflammatory marker.

A differential association between SES groups in terms of personality and health-related factors may have implications for health promotion and intervention [12]. A strategy of adapting interventions to the behaviours and beliefs that characterise particular personality types may improve the implementation of intervention programmes. There have been cogent proposals that interventions targeted at low SES groups may be refined to account for dispositional differences [31], and that the identification of individuals whose personality styles render them vulnerable to particular health risks would allow them to benefit from closer monitoring which might result in earlier detection and treatment [45]. Our findings confirm that account should be taken of SES when exploring relationships between personality and biological factors relating to health: indeed, the overall main effect of N gives the misleading impression of a positive relationship between neuroticism and CRP and IL-6 across the sample. In fact, the relationship may be reduced or reversed in deprived individuals, confirming proposals that the association between personality and inflammation may differ amongst population sub-groups [21], [31] and implying that targeted interventions may require particular subtlety.

The present study has a number of limitations. The most significant is the cross-sectional design which precludes any attribution of causality. The three-factor assessment of personality precluded analysis of some facets of N and E that may determine the expression, or inhibition, of particular health-related behaviours [6]. The sample size, while large by comparison with some other studies, was determined by the numbers needed to detect between-group differences in two biological variables but, as the effect sizes due to personality variables are typically small [13], the sample size may have been insufficient to detect subtle effects of personality at high levels of inflammation. Moreover, whilst the sampling procedure was successful in recruiting two samples which differed widely in terms of SES, their composition was, self-evidently, not fully representative of the Scottish population. These considerations lead to the more general question as to whether the present results are representative of “deprived” individuals within the population. We acknowledge that our MD sample may not represent an extreme of socio-economic deprivation for the simple reason that such individuals do not readily volunteer as research subjects [49]. We therefore concede that the present MD subjects may represent a sub-group whose relative intellect and years of education have encouraged an interest in health and well-being, and provided the confidence to volunteer and engage in the study. The outgoing and sociable characteristics of those higher in E would further encourage this tendency. Finally, the exclusively white Caucasian sample also precluded examination of ethnic differences that have been shown relevant in other investigations of personality and inflammation [21]. We also note the high number of statistical tests performed, which might have resulted in an increased type 1 error rate.

Despite these limitations, the demonstration of an interaction between personality, deprivation and inflammation is a novel finding. Evidence that the expression of personality influences upon inflammation may differ within sub-groups of the population provides a caveat that misleading conclusions may be drawn as to the relationship between personality and biological variables if the factor of SES is neglected.
